# A gross deletion of the *PAX3* gene in a large Chinese family with Waardenburg syndrome type I

**DOI:** 10.1007/s12519-023-00746-2

**Published:** 2023-09-14

**Authors:** Shu-Zhi Yang, Lei Hou, Xin Qi, Guo-Jian Wang, Sha-Sha Huang, Shan-Shan Zhang, Bang-Qing Huang, Ying Yang, Bei-Cheng Li, Shuo Liu, Pu Dai, Yu Su

**Affiliations:** 1 Department of Otolaryngology, The 6th Medical Center of Chinese PLA General Hospital, No. 6, Fucheng Road, Haidian District, Beijing 100048, China; 2 National Clinical Research Center for Otorhinolaryngologic Disease, Chinese PLA General Hospital, No.6, Fucheng Road, Haidian District, Beijing 100048, China; 3 Department of Otolaryngology, Hainan Hospital Affiliated to Chinese PLA General Hospital, Jianglin Road, Haitang District, Sanya 572013, China; 4 Department of Neurology, The First Medical Center of Chinese PLA General Hospital, No. 28, Fuxing Road, Haidian District, Beijing 100853, China

Waardenburg syndrome (WS) is an autosomal-dominant neurocristopathy characterized by sensorineural hearing loss (SNHL) and pigmentary abnormalities of the iris, hair, and skin. It is responsible for approximately 3% of congenital hearing loss [1]. WS is thought to be fully penetrant when considering at least one clinical feature, but the penetrance of each feature is not complete [2]. Four types of WS have been classified based on the clinical manifestations. WS1 is one of the most common forms of WS. To confirm the diagnosis of WS1, at least two major or one major plus two minors of the Waardenburg consortium criteria must be present. In brief, major criteria include congenital sensorineural hearing loss, pigmentary disturbances of the iris, hair hypopigmentation, dystopia canthorum (average W index of affected family members is greater than 1.95) and affected first-degree relatives. Minor criteria include skin hypopigmentation, synophyrys or medial eyebrow flare, broad and high nasal root, hypoplasia of alae nasi, and premature graying of hair [3]. High phenotypic heterogeneity has been observed in WS1, even in the same family [4]. Pathogenic variants of the *PAX3* gene have been identified in more than 90% of affected individuals with WS1. *PAX3* encodes paired box gene 3, a transcription factor in neural crest cells of the spinal ganglia, the craniofacial mesectoderm, and the limb mesenchyme during embryogenesis. It plays an important role in the migration and differentiation of melanocytes, which originate from the embryonic neural crest. To date, at least 149 pathogenic variants of the *PAX3* gene have been identified in WS1 or WS3, the majority of which are missense or nonsense pathogenic variants (75/149) [5], while 15 gross deletions have been reported by multiplex ligation-dependent probe amplification (MLPA) or fluorescent in situ hybridization. In this study, targeted next-generation sequencing (NGS) technology was employed to reveal the molecular cause of WS1 in a large Chinese family.

The proband (III:7) was a 1-year 11-month-old girl who presented at birth with dystopia canthorum, white forelock and bilateral brilliant blue irides. She was admitted to the hospital for cochlear implantation. She failed her initial and follow-up newborn hearing screening at 72 hours and 42 days. Her parents reported that she had no response to sound at 1 year old. Further hearing tests were conducted in our hospital to verify the diagnosis of bilateral profound hearing loss. According to the criteria for WS1 proposed by the Waardenburg consortium, she was diagnosed with WS1 [3]. Intriguingly, she also presented with thumb polydactyly on her left hand, in which the chief thumb had normal shape and function, and the second-floating thumb had no bony or articular relationship with the chief thumb. This was diagnosed as type I according to the Wassel classification, which is used to classify preaxial polydactyly based on the level of duplication from distal to proximal [6]. The patient was a sporadic case, and no other family members manifested polydactyly.

A comprehensive questionnaire was administered to the child’s parents and revealed a four-generation WS1 family (Fig. 1). In total, there were 31 family members in this family. Fifteen individuals completed a comprehensive clinical history with neurotological, ophthalmological, dermatological and audiological assessments. Twelve of the 15 family members consented to provide blood samples for genotyping.

**Fig. 1:**
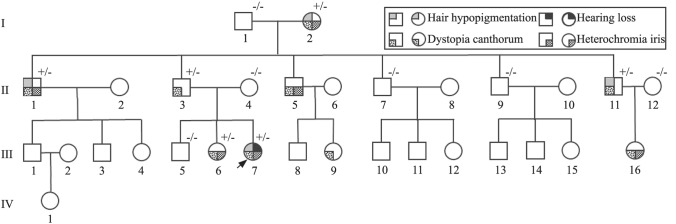
The pedigree of the Waardenburg syndrome type I family shows high phenotypic heterogeneity, with not all affected cases exhibiting all features of the syndrome. Dystopia canthorum was the most common phenotype observed in this family. The upper right corner marks the genotype. “+/−” heterozygous mutation, “−/−” negative

Dystopia canthorum was the most common phenotype present in all nine affected family members (9/9, 100.0%). The average W index of all nine affected individuals was 2.78, which exceeds the clinical diagnostic criteria of 1.95 for WS1 proposed by the WS consortium [5]. Pigmentary disturbances of the iris were the second most common phenotype presented in six out of nine participants (6/9, 66.7%, Fig. 2). Among the six individuals who manifested pigmentary disturbances of the iris, three had bilaterally brilliant blue irises, and three had complete heterochromia iridium (one iris was brown, and another iris was blue). No partial heterochromia iridis was found in this study. Although no abnormal fundus results were detected by direct ophthalmoscopy, abnormalities of choroidal pigmentation (particularly abnormalities in the peripheral fundus) could have been missed by direct ophthalmoscopy. Moreover, no visual impairments were observed in the affected individuals. Hypopigmentation of hair in the form of white forelock was the third major phenotype present in four of nine participants (4/9, 44.4%). Premature graying hair, another abnormality of pigmentary distribution, was observed in this pedigree and present in three out of nine participants (3/9, 33.3%). Three cases manifested white forelock and grabbing hair concurrently. Several spotted areas of hypopigmentation of the skin were found on the trunk or limbs in four cases (4/9, 44.4%). Synophrys was found in III:16 only. SNHL was observed only in the proband, who showed congenital, bilateral profound hearing loss. High-resolution computed tomography of the temporal bone revealed a normal anatomy. Cochlear implantation was successfully performed (Nurotron CS-10A, China), and satisfactory auditory performance was achieved after cochlear implantation. Detailed clinical features of this WS1 family can be found in Fig. 2 and Table 1.

**Fig. 2:**
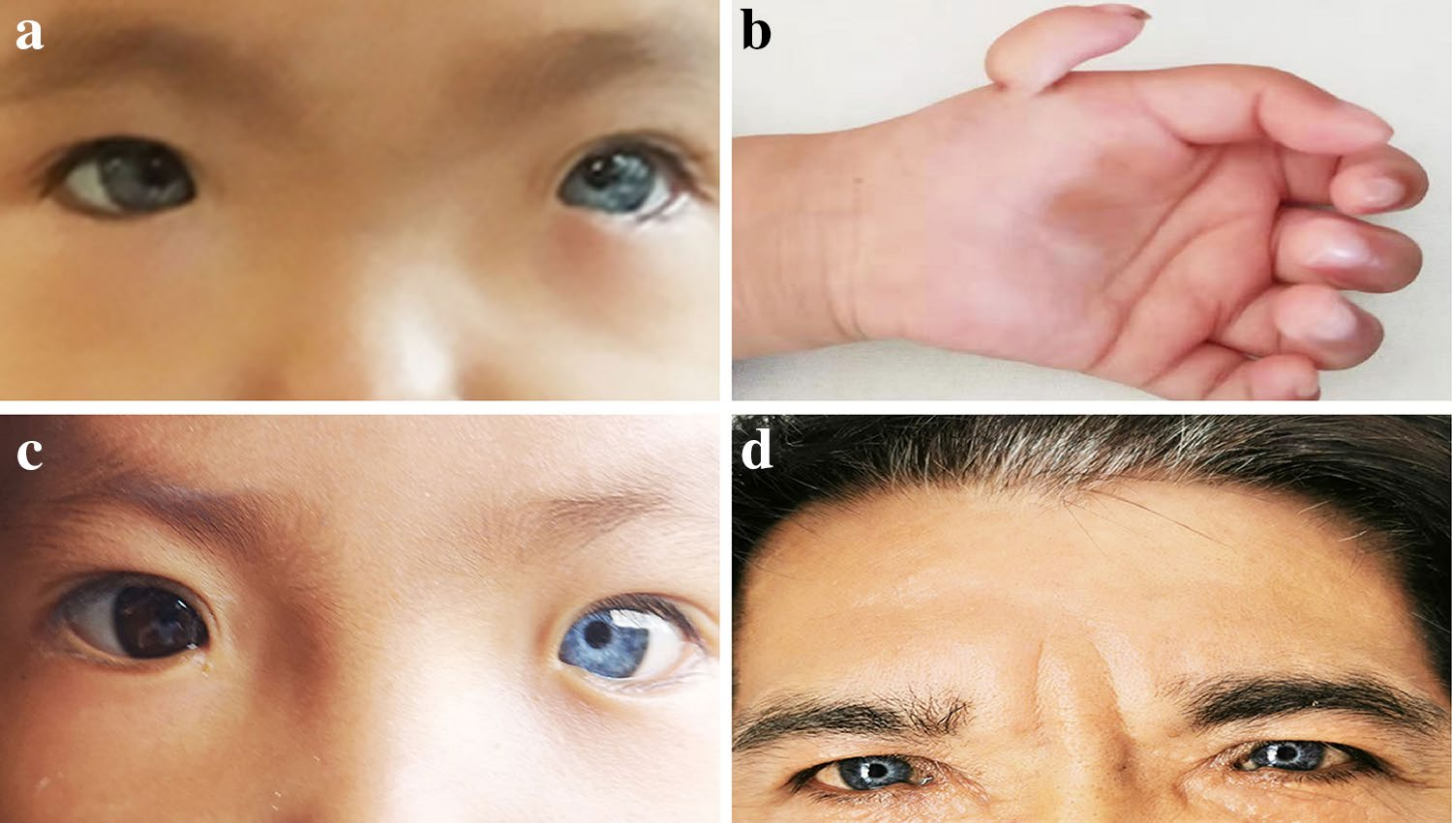
The major clinical features in this family. **a** Proband III:7 manifested dystopia canthorum, broad nasal root, and bilateral brilliant blue irides; **b** proband III:7 showed thumb polydactyly on left hand, with the chief thumb having a normal shape and function, and the second-floating-thumb having no bony or articular relationship with the chief thumb; **c** individual III:6 exhibited dystopia canthorum, broad nasal root, and complete heterochromia iridum; **d** individual II:1 had a white forelock and bilateral brilliant blue iridia

**Table 1:** Main clinical features of the nine affected family members of WS1 pedigree

No	Dystopia canthorum	Pigmentary disturbances of iris	Hypopigmentation of hair	Skin spots	Synophrys	Hearing loss
I:2	+	A	+	+	−	−
II:1	+	B	+	−	−	−
II:3	+	−	−	−	−	−
II:5	+	B	−	−	−	−
II:11	+	−	+	−	−	−
III:6	+	A	−	+	−	−
III:7	+	B	+	+	−	+
III:9	+	−	**−**	+	−	−
III:16	+	A	**−**	−	+	−

*A* complete heterochromia iridum, *B* brilliant blue iris. “+” sign present, “−” sign absent

Target NGS technology detected a complete deletion of the coding sequence of the *PAX3* gene in the proband. The deletion was annotated as likely pathogenic, which included PVS1 + PM2 supporting evidence. MLPA results verified heterozygous deletion of exons 1 to 8 of *PAX3* in the proband’s sample (Fig. 3). The mutation was also detected in five other affected family members who provided DNA samples (I:2, II:1, II:3, II:11, and III:6). Furthermore, unaffected family members tested negative for this mutation.

**Fig. 3:**
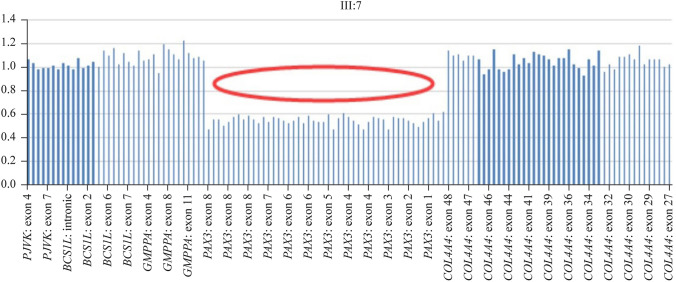
Target next-generation sequencing technology identified a complete deletion of the coding sequence of the *PAX3* gene in the proband’s sample. *PJVK* pejvakin gene, *BCS1L* Bc1 synthesis-like gene, *GMPPA* guanosine diphosphate-mannose pyrophosphorylase A gene, *COL4A4* collagen type IV alpha 4 chain gene, *PAX3* paired box gene 3

In conclusion, we report the results of a study on a large Chinese family with WS1. High clinical variability was observed in this family. Dystopia canthorum is the most penetrant feature of WS1 in this family; pigmentary disturbance of the iris is the second most common phenotype. These two clinical features are easier to identify at birth, so they may imply an early diagnosis of WS. SNHL is a well-known feature of WS1 with a reported 60% penetrance [2]. However, in this family, SNHL had a much lower penetrance, with only one case affected. The cause of the lower penetrance of SNHL is unclear, but genetic modification and environmental factors may contribute to this difference.

WS3 and WS1 have very similar features but are distinguished by musculoskeletal anomalies, which are only present in WS3. Patients with WS3 present with flexion contractures and muscle hypoplasia of the upper limbs with a broad range of severity [1]. Heterozygous *PAX3* mutations are expected to be responsible for most WS1, and homozygous or compound heterozygous *PAX3* mutations have been described in severe cases of WS3 [5]. In this family, although the proband manifested polydactyly on her left hand, no other family members manifested polydactyly. Furthermore, a heterozygous *PAX3* mutation was detected, so we thought the patient was a sporadic case of polydactyly and diagnosed with WS1.

Heterozygous pathogenic variants in *PAX3* are the main cause of WS1. To date, almost 149 different *PAX3* pathogenic variants have been reported in association with WS1, with about half of them being missense/nonsense mutations and only a few being recurrent. Partial or total gene deletions have also been described and may account for up to 10% of cases in which no point mutations have been identified [4]. In this pedigree, a novel gross deletion of the *PAX3* gene (deletion of exons 1 to 8 in heterozygous status) was identified and segregated with disease. This deletion was annotated as likely pathogenic and was supported by PVS1 + PM2 evidence. Although this mutation causes a complete loss of *PAX3* gene function, the pathogenic effect needs to be confirmed by functional studies.

A thorough review of the literature revealed that 8 papers have reported partial or whole *PAX3* deletions in WS1 patients, and 7 out of 8 papers described a total of 16 cases with available clinical data [7–14]. Dystopia canthorum had complete penetrance in all 16 cases. Heterochromia irises were observed in 10 cases (5 with brilliant blue irises and 5 with complete heterochromia irises), while hearing loss was manifested in 10 patients. In this family, dystopia canthorum and heterochromia iris have the same situation, but hearing loss has lower penetrance. To date, there has been no correlation between the mutation type (whole or partial deletion) and the severity of the phenotype. Therefore, loss of protein function leading to haploinsufficiency seems to be the disease-causing mechanism for WS1 [15].

Human genetic variants, comprising single-nucleotide variants (SNVs), small insertions or deletions, and structural variants (SVs), contribute to many physical traits and human diseases. Sanger sequencing, known as the gold standard of sequencing technology, is a powerful tool for identifying SNVs of *PAX3* but not SVs. This is because SVs, such as copy number variations and sequence losses, are highly polymorphic and widely distributed in the genome [16]. Therefore, detecting SVs presents significant challenges. MLPA and NGS are traditional detection methods for identifying SVs. This is the first report of a gross deletion of the *PAX3* gene identified by NGS in Chinese WS patients. Our findings demonstrate that NGS is an effective instrument for detecting large deletions in *PAX3* and increasing the sensitivity of genetic diagnosis.

## Data Availability

The datasets are available from the corresponding author on reasonable request.
